# Alterations of plasma metabolomes and their correlations with immunogenicity in maintenance hemodialysis patients receiving different COVID‐19 vaccine regimens

**DOI:** 10.14814/phy2.70005

**Published:** 2024-08-19

**Authors:** Phoom Narongkiatikhun, Chanisa Thonusin, Sirawit Sriwichaiin, Wichwara Nawara, Kanda Fanhchaksai, Nuttanun Wongsarikan, Sirinart Kumfu, Nipon Chattipakorn, Siriporn C. Chattipakorn

**Affiliations:** ^1^ Division of Nephrology, Department of Internal Medicine, Faculty of Medicine Chiang Mai University Chiang Mai Thailand; ^2^ Cardiac Electrophysiology Unit, Department of Physiology, Faculty of Medicine Chiang Mai University Chiang Mai Thailand; ^3^ Cardiac Electrophysiology Research and Training Center, Faculty of Medicine Chiang Mai University Chiang Mai Thailand; ^4^ Center of Excellence in Cardiac Electrophysiology Research Chiang Mai University Chiang Mai Thailand; ^5^ Division of Hematology and Oncology, Department of Pediatrics, Faculty of Medicine Chiang Mai University Chiang Mai Thailand; ^6^ Department of Internal Medicine, Faculty of Medicine Chiang Mai University Chiang Mai Thailand; ^7^ Department of Oral Biology and Diagnostic Sciences, Faculty of Dentistry Chiang Mai University Chiang Mai Thailand

**Keywords:** COVID‐19, hemodialysis, plasma metabolomes, vaccine, vaccine immunogenicity

## Abstract

Maintenance hemodialysis (MHD) patients exhibit compromised immune responses, leading to lower immunogenicity to the COVID‐19 vaccine than the general population. The metabolomic factors influencing COVID‐19 vaccine response in MHD patients remain elusive. A cross‐sectional study was conducted with 30 MHD patients, divided into three vaccine regimen groups (*N*= 10 per group): homologous CoronaVac^®^ (SV‐SV), homologous ChAdOx1 nCoV‐19 (AZ‐AZ), and heterologous prime‐boost (SV‐AZ). Plasma samples were collected at baseline and at 28 days after the final dose to analyze 92 metabolomic levels using targeted metabolomics. The study included 30 MHD patients (mean age 56.67 ± 10.79 years) with similar neutralizing antibody (nAb) levels across vaccine regimens. The most significant differences in metabolomics were found between AZ‐AZ and SV‐SV, followed by SV‐AZ versus SV‐SV, and AZ‐AZ versus SV‐AZ. Overall, the metabolomic changes involved amino acids like glutamate and phenylalanine, and phospholipids. Prevaccination metabolomic profiles, including PG (38:1), lysoPE (20:2), lysoPC (18:2), lysoPI (18:1), and PC (34:2), exhibited negative correlations with postvaccination nAb levels. Different COVID‐19 vaccine regimens had unique interactions with the immune response in MHD patients. Amino acid and phospholipid metabolisms play crucial roles in nAb formation, with the phospholipid metabolism being a potentially predictive marker of vaccine immunogenicity among MHD patients.

## 1 INTRODUCTION

The coronavirus disease 2019 (COVID‐19) pandemic posed an unprecedented global health challenge, with severe consequences for vulnerable populations, including individuals undergoing hemodialysis (Clift et al., 2020). Maintenance hemodialysis (MHD) patients often exhibit compromised immune responses, rendering them particularly susceptible to severe manifestations of COVID‐19 (El Karoui & De Vriese, 2022). In response to this heightened risk, various vaccines have been developed and deployed to mitigate the impact of the virus on this at‐risk demographic.

Vaccine immunogenicity is defined as the ability of a vaccine to stimulate an immune response in the vaccinated individual (Banaszkiewicz & Radzikowski, 2013). Although previous evidence indicated a reduction in the immune response to vaccinations in MHD patients (El Karoui & De Vriese, 2022), various strategic approaches have been implemented, involving either elevated vaccine doses or an increased frequency of vaccine administration in those patients (Liao et al., 2016; Mulley et al., 2017). However, other factors, in addition to vaccine efficacy, play a pivotal role in influencing immunogenicity. Previous data indicated that intrinsic host factors, behavioral aspects, environmental conditions, nutritional considerations, perinatal host factors, and baseline immunity collectively constituted potential determinants impacting both vaccine immunogenicity and its overall efficacy (Zimmermann & Curtis, 2019).

Metabolomics has emerged as a pivotal field among the various omics disciplines, offering a valuable tool for evaluating the presence and role of small molecules within a given specimen (Gomase et al., 2008). The integration of metabolomics with systems biology, including transcriptomics, and proteomics, presents unique opportunities to bridge existing gaps in our understanding of the pathogenesis, diagnosis, prevention, and potential treatment of multiple diseases (Petersen et al., 2013). Furthermore, the judicious application of metabolomics within the field of vaccinology holds promise in elucidating the intricate details of metabolomes associated with vaccine immunogenicity. This field not only enhances our comprehension of vaccine responses, but also facilitates the formulation of tailored vaccine regimens specifically designed for distinct populations (Borriello et al., 2018). However, evidence of changes in plasma metabolomes and their correlations with SARS‐CoV‐2 vaccine immunogenicity in MHD patients remains limited.

This study aimed to assess changes in plasma metabolomes following different regimens of SARS‐CoV‐2 vaccinations in MHD patients. We also aimed to investigate pre‐ and postvaccination metabolomics associated with the vaccine immunogenicity to enhance our understanding of the molecular basis of COVID‐19 vaccination in MHD patients. To achieve our objectives, we utilized residual plasma samples from the project “Immunogenicity and Safety of Homologous and Heterologous Prime‐Boost of CoronaVac® and ChAdOx1 nCoV‐19 in Hemodialysis Patients: An Observational Prospective Cohort Study.”

## 2 MATERIALS AND METHODS

### 2.1 Study approval

All participants were enrolled onto a prospective study (TCTR20220516002). The study received approval from the local Institutional Review Board of the Faculty of Medicine, Chiang Mai University, Chiang Mai, Thailand (protocol code: MED‐2566‐0187, date of approval: September 29^th^ 2023), in adherence with the Declaration of Helsinki. All participating individuals provided written informed consent prior to enrolment onto the study.

### 2.2 Immunogenicity and safety of homologous and heterologous prime‐boost of CoronaVac® and ChAdOx1 nCoV‐19 among hemodialysis patients: An observational prospective cohort study

The study protocol was continued from our previous study (Narongkiatikhun et al., 2023). This previous trial enrolled 130 MHD patients who had been in a stable condition for at least 3 months prior to enrollment. These patients intended to receive one of the following vaccine regimens: homologous inactivated CoronaVac® vaccine (Sinovac, Beijing, China) against SARS‐CoV‐2 (SV‐SV), homologous replication‐defective viral vectors ChAdOx1 nCoV‐19 vaccine (AZD1222) against SARS‐CoV‐2 (AZ‐AZ), or the heterologous prime‐boost of inactivated vaccine followed by the replication‐defective viral vectors vaccine (SV‐AZ). Details of each vaccine regimen were described in the previous study (Narongkiatikhun et al., 2023). The immunogenicity of the SARS‐CoV‐2 vaccination was evaluated one to 3 days before the first vaccination as the baseline level, and on 28 days (4 weeks) after completing the vaccination regimens. This specific assessment period was based on a previous study that showed antibody levels peaked at 28 days postvaccination (Amellal et al., 2023). Additionally, several studies on vaccine immunogenicity support that a 4‐week interval after vaccination is reliable for assessing the immune response (Amellal et al., 2023; Puspitasari et al., 2023; Yau et al., 2021).

In determining the sample size for each vaccine regimen, we followed the stepped rules of thumb based on pilot trial data (Lewis et al., 2021). For a medium effect size and an 80% power, we calculated that 10 participants per vaccine regimen group would be necessary. Consequently, left‐over plasma samples from 30 patients out of 130 participants from a previous study were selected. To ensure representativeness, patients in each vaccine regimen group were chosen based on matching demographic parameters, including age (categorized as <55 and ≥ 55 years) and gender, considering the diverse factors influencing metabolomics. A summary of the protocol is shown in Figure S1.

### 2.3 Evaluation of humoral response by using SARS‐CoV‐2 surrogate virus neutralization test (sVNT)

Humoral immunity (HMI) was evaluated using the SARS‐CoV‐2 NeutraLISA surrogate neutralization assay (Euroimmun, Germany, catalogue number EI 2606–9601‐4), which reflects the neutralizing antibody (nAb) levels—an indicator showing a high correlation with immune protection. In accordance with the manufacturer's instructions, a cutoff value of ≥35% inhibition (IH) was indicative of seroconversion, while values ≥20–<35% IH and <20% IH were categorized as borderline and negative, respectively. There were no alterations or additions made to the human plasma prior to the assessment of the vaccine response. The plasma samples were collected, processed, and stored following standard protocols to maintain their integrity and avoid any potential interference with the assessment of vaccine immunogenicity.

### 2.4 Plasma samples and the preparation for targeted metabolomics

Plasma samples from the previous mentioned cohort were stored at −80°C until the extraction process. To precipitate proteins and extract plasma metabolomes, 200 μL of extraction solvent (1:1:1 methanol: acetonitrile: acetone) was added to 50 μL of plasma. The samples were vortexed for 10 s, allowed to rest on ice for 5 min, and then centrifuged at 16,000 *g* for 10 min at 4°C. The supernatant was then transferred to an autosampler vial for liquid chromatography‐mass spectrometry (LC–MS) analysis using an Agilent 1290 infinity II LC with an Agilent 6546 quadrupole time‐of‐flight MS (Santa Clara, CA).

### 2.5 Targeted metabolomics study by mass spectrometry

The steps of plasma metabolome extraction were detailed in prior studies (Thonusin et al., 2023). Then, each sample was transferred to an autosampler vial for liquid chromatography‐mass spectrometry (LC–MS) analysis using an Agilent 1290 infinity II LC with an Agilent 6546 quadrupole time‐of‐flight MS (Santa Clara, CA). Three LC–MS analyses were performed per sample: negative ion mode hydrophilic interaction chromatography—electrospray ionization mass spectrometry (HILIC‐ESI‐MS) and both positive and negative ion mode reversed phase liquid chromatography‐electrospray ionization mass spectrometry (RPLC‐ESI‐MS). These approaches were used to enable the detection of amino acids, free fatty acids, bile acids, acylcarnitines, and phospholipids (Thonusin et al., 2017). All of these metabolomes have previously been shown to strongly indicate the metabolic signatures of vaccine‐induced responses (Diray‐Arce et al., 2020). The details of HILIC and RPLC, along with MS parameters and metabolome quantification have been described in previous studies. (Thonusin et al., 2017).

### 2.6 Plasma metabolome quantification

Each plasma metabolome was quantified using Agilent MassHunter Quantitative Analysis Software version 10.1 (Agilent technologies, Santa Clara, CA, USA). Then, the quantitated peak areas of plasma metabolomes with external standards (both with and without stable isotope labeled internal standards) were converted to concentrations in accordance with the calibration curve of each plasma metabolome. Thereafter, peak areas and concentrations of plasma metabolomes with no stable isotope labeled internal standards were normalized under a normalization tool for multibatch metabolomic data as described in a previous study (Thonusin et al., 2017).

### 2.7 Study outcomes

The primary objective of this study was to determine the variations in metabolomic alterations among different COVID‐19 vaccine regimens (SV‐SV, AZ‐AZ, and SV‐AZ) in MHD patients. The secondary objectives included the identification of plasma metabolomes, both pre‐ and post‐COVID‐19 vaccination, that correlated with nAb levels.

### 2.8 Statistical analysis

The statistical analyses were conducted on the Metaboanalyst 5.0 platform (Pang et al., 2022) and the data visualization was conducted by Metaboanalyst 5.0 and R version 4.1.1.

Firstly, to demonstrate the differences in the alteration of plasma metabolomes among vaccination regimens, the comparisons of each plasma metabolome between two vaccination regimens were conducted after Log 10 transformation and Pareto scaling of metabolite concentration. One‐way analysis of variance (one‐way ANOVA) with post hoc LSD was used for the comparison. The violin plot was used for demonstration of the significantly different plasma metabolomes in each pair of comparisons. A *p*‐value of less than 0.05 was considered as a threshold for statistical significance.

Secondly, the correlations between plasma metabolomes and nAb at the last visit of participants were investigated and demonstrated using Pearson's correlation. A *p*‐value of less than 0.05 was considered as a threshold for statistical significance. Scatter plots were used to demonstrate the significant correlations and bar plots were used to demonstrate the top 25 plasma metabolomes correlated with nAb.

Thirdly, the correlations between prevaccination plasma metabolomes and postvaccination nAb were investigated. Pearson's correlations were determined, and scatter plots and bar plots were used for data visualization as mentioned above. A *p*‐value of less than 0.05 was considered as a threshold for statistical significance.

## 3 RESULTS

### 3.1 Baseline characteristics between each regimen of vaccination

A total of 30 MHD patients were selected with careful age and gender matching for each regimen of vaccinations. The baseline characteristics of the study were categorized into three regimens of COVID‐19 vaccine: AZ‐AZ, SV‐AZ, and SV‐SV, with 10 participants in each group (Table 1). The cohort had a mean age of 56.67 ± 10.79 years old and a balanced gender distribution. The mean body mass index (BMI) was 24.24 ± 6.14 kg/m^2^. Smoking prevalence was 76.67%, with no significant differences between regimens. Common comorbidities including hypertension (93.33%), dyslipidemia (40.00%), and type 2 diabetes mellitus (36.67%) were not significantly different between groups. Dialysis vintage significantly varied between different regimens (median [min, max] 43.5 [26, 48] months, 92 [36, 147] months, and 29 [22, 72] months for AZ‐AZ, SV‐AZ, and SV‐SV, respectively; *p* = 0.024). Overall, laboratory results showed similar findings between the groups (Table 1).

**TABLE 1:** Baseline characteristics of maintenance hemodialysis patients classified by vaccine regimens.

Variable	Total (*N* = 30)^a^	AZ‐AZ (*n* = 10)^a^	SV‐AZ (*N* = 10)^a^	SV‐SV (*N* = 10)^a^	*p*‐Value
Age—years *n* (mean)					
<55	13 (43.33)	3 (30)	3 (30)	7 (70)	0.114
≥55	17 (56.67)	7 (70)	7 (70)	3 (30)	
Female	4 (40)	4 (40)	4 (40)	4 (40)	1.000
Body mass index—kg/m^2^	24.24 ± 6.14^b^	24.27 ± 4.01^b^	21.08 ± 3.57^b^	27.38 ± 8.39^b^	0.066
Smoking (units)					
No	23 (76.67)	8 (80)	6 (60)	9 (90)	0.479
Active smoker	2 (6.67)	0 (0)	2 (20.00)	0 (0)	
Exsmoker	5 (16.67)	2 (20)	2 (20)	1 (10)	
Comorbid disease (units)					
Hypertension	28 (93.33)	9 (90)	9 (90)	10 (100)	1.000
Dyslipidemia	12 (40)	4 (40)	6 (60)	2 (20)	0.189
Diabetes mellitus	11 (36.67)	5 (50)	4 (40)	2 (20)	0.510
Cardiovascular disease (MI, HF)	4 (13.33)	2 (20)	1 (10)	1 (10)	1.000
Cerebrovascular disease	1 (3.33)	0 (0)	0 (0)	1 (10)	1.000
Chronic obstructive pulmonary disease	1 (3.33)	0 (0)	1 (10)	0 (0)	1.000
Connective tissue disease	1 (3.33)	0 (0)	1 (10)	0 (0)	1.000
Liver disease	2 (6.67)	1 (10)	1 (10)	0 (0)	1.000
Cause of End‐stage kidney disease (units)					
Diabetic kidney disease	9 (30)	4 (40)	4 (40)	1 (10)	0.090
Hypertensive nephropathy	3 (10)	1 (10)	0 (0)	2 (20)	
Obstructive uropathy	4 (13.33)	0 (0)	3 (30)	1 (10)	
Glomerular disease	9 (30)	3 (30)	1 (10)	5 (50)	
Others causes	2 (6.67)	2 (20)	0 (0)	0 (0)	
Unknown cause	3 (10)	0 (0)	2 (20)	1 (10)	
Dialysis vintage—months	45.5 (26, 72)^c^	43.5 (26, 48)^c^	92 (36, 147)^c^	29 (22, 72)^c^	0.024
Dialysis schedule					
1–2 time/week	6 (20)	2 (20)	2 (20)	2 (20)	1.000
3–4 time/week	24 (80)	8 (80)	8 (80)	8 (80)	
Mode of hemodialysis					
Conventional hemodialysis	18 (93.33)	10 (100)	10 (100)	8 (80)	0.310
Online hemodiafiltration	2 (6.67)	0 (0)	0 (0)	2 (20)	
Urine output:					
No	12 (40)	5 (50)	3 (30)	4 (40)	0.659
Yes	18 (60)	5 (50)	7 (70)	6 (60)	
Laboratory results					
Hb—g/dL	10.25 ± 1.83^b^	10.43 ± 1.98^b^	9.87 ± 2.24^b^	10.44 ± 1.27^b^	0.742
<10	11 (36.67)	2 (20)	4 (40)	5 (50)	0.366
≥10	19 (63.33)	8 (80)	6 (60)	5 (50)	
White blood cells—cells/mm^3^	6859 ± 2351.34^b^	6216.00 ± 1834.50^b^	7993.00 ± 3261.63^b^	6368.00 ± 1286.59^b^	0.175
Polymorphonuclear cell—%	63.93 ± 9.99^b^	63.08 ± 6.49^b^	67.75 ± 13.26^b^	60.96 ± 8.76^b^	0.309
Lymphocyte—%	21.69 ± 4.89^b^	23.33 ± 3.47^b^	18.90 ± 6.53^b^	22.85 ± 3.06^b^	0.080
Platelet— × 10^3^ cells/mm^3^	211,100 ± 62964.88^b^	210,100 ± 86362.86^b^	218,700 ± 62453.63^b^	204,500 ± 36084.01^b^	0.886
C‐reactive protein—mg/L	3.12 (3.12, 6.04)^c^	3.12 (3.12, 8.66)^c^	3.465 (3.12, 6.94)^c^	3.12 (3.12, 5.96)^c^	0.863
Albumin—g/dL	4.13 ± 0.30^b^	4.09 ± 0.23^b^	4.11 ± 0.26^b^	4.18 ± 0.41^b^	0.793
Kt/v	1.57 ± 0.39^b^	1.46 ± 0.26^b^	1.74 ± 0.55^b^	1.51 ± 0.27^b^	0.230

^a^ Number (%).

^b^ Mean ± SD.

^c^ Median (min, max).

Abbreviations: AZ‐AZ, homologous AZD1222 regimen; HF, heart failure; MI, myocardial infarction; SV‐AZ, heterologous Sinovac‐AZD1222 regimen; SV‐SV, homologous Sinovac regimen.

### 3.2 Percentage of neutralizing antibody after complete course of each regimen of vaccinations

Twenty‐eight days after the completion of each regimen, we measured nAb. The AZ‐AZ group showed a median percentage (min, max) of 73.75 (33.22, 99.25), while SV‐AZ and SV‐SV demonstrated 73.21 (−8.25, 99.18) and 50.08 (−1.34, 80.15), respectively (*p* = 0.133).

### 3.3 Metabolomics analysis

**FIGURE 1:**
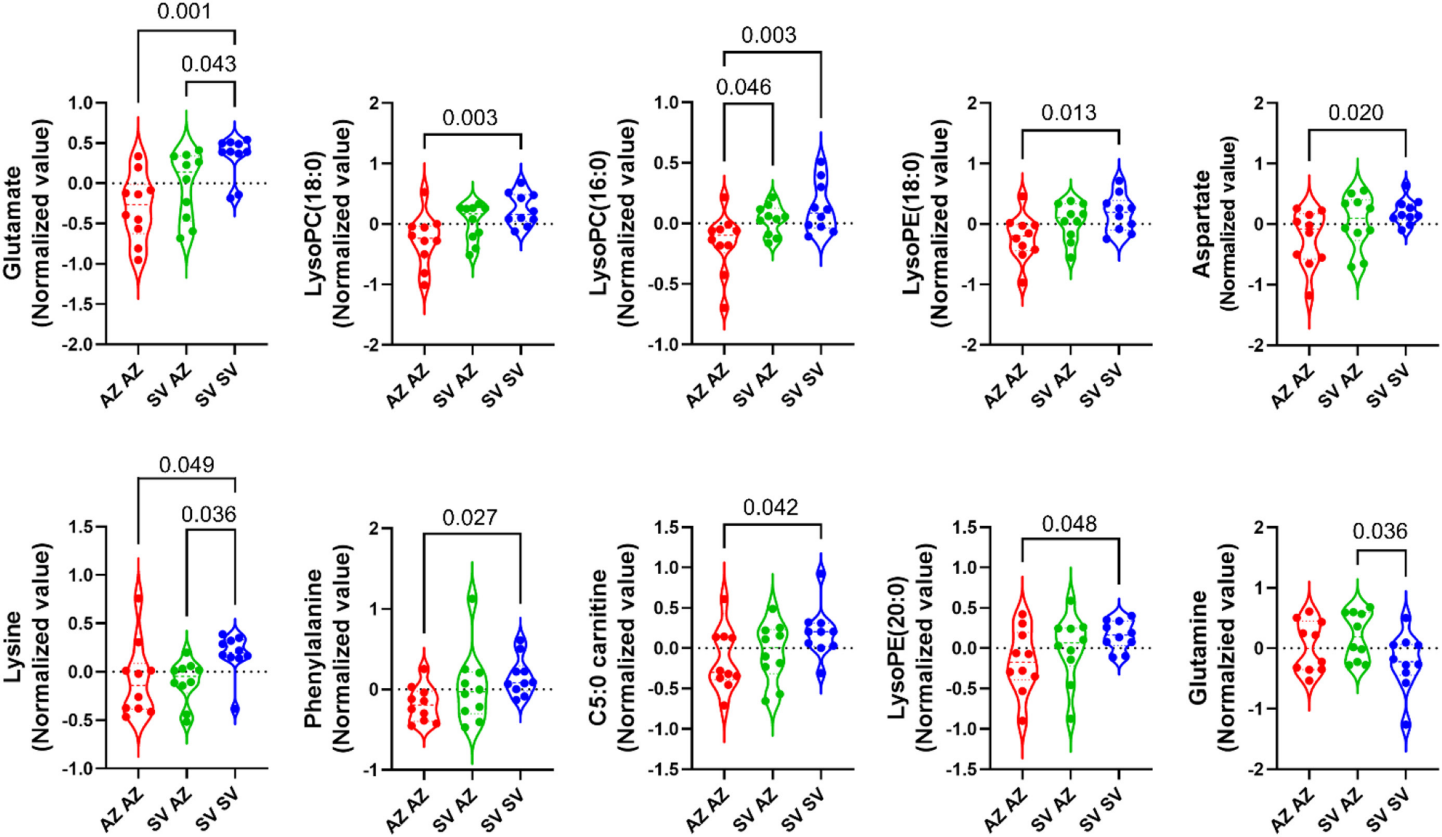
Comparisons of plasma metabolomes after complete vaccination for each pair of regimens: AZ‐AZ (homologous AZD1222), SV‐AZ (heterologous Sinovac‐AZD1222), and SV‐SV (homologous Sinovac).

Prevaccination, plasma metabolomes between each vaccine regimens were similar **(**This article contains supplemental data, Figure S2). At day twenty‐eight after complete course of vaccination, four plasma metabolomes, including glutamate (*F* = 7.040, ANOVA *p*‐value = 0.003), lysoPC (18:0) (5.372, 0.011), lysoPC (16:0) (5.311, 0.011), and lysoPE (18:0) (3.690, 0.038), showed different changes between each of the vaccine regimens (ANOVA *p*‐value < 0.05) **(**Table 2 **)**. Principal component analyses of plasma metabolomes are shown in Figure S3. Pairwise comparisons of plasma metabolomes after a complete course of vaccination between each pair of vaccination regimens were also conducted (Figure 1). The comparisons between the AZ‐AZ and SV‐SV regimens demonstrated that some plasma metabolomes were down‐regulated in the AZ‐AZ group, including glutamate (LSD adjusted *p*‐value < 0.001), phenylalanine (0.027), lysoPC (18:0) (0.003), lysoPC (16:0) (0.003), aspartate (0.012), lysoPE (18:0) (0.013), lysoPE (20:0) (0.048), lysine (0.049), and C5:0 carnitine (0.042). On the other hand, the comparison between SV‐AZ versus SV‐SV and AZ‐AZ versus SV‐AZ regimens showed only a few different plasma metabolomes, including down‐regulation of lysine (0.036) and glutamate (0.043), and upregulation of glutamine (0.036) in SZ‐AZ when compared to SV‐SV, and down‐regulation of lysoPC (16:0) (0.046) in AZ‐AZ when compared to SV‐AZ.

**TABLE 2:** Comparison of plasma metabolomes on day twenty‐eight after complete vaccination between each vaccine regimens.

Plasma metabolomes	*F*‐value	*p*‐Value
Glutamate	7.040	0.003
LysoPC (18:0)	5.372	0.011
LysoPC (16:0)	5.312	0.011
LysoPE (18:0)	3.690	0.038
Aspartate	3.129	0.060
Lysine	3.040	0.064
Phenylalanine	2.773	0.080
Glutamine	2.466	0.104
C5:0 carnitine	2.420	0.108
LysoPE (20:0)	2.149	0.136

**FIGURE 2:**
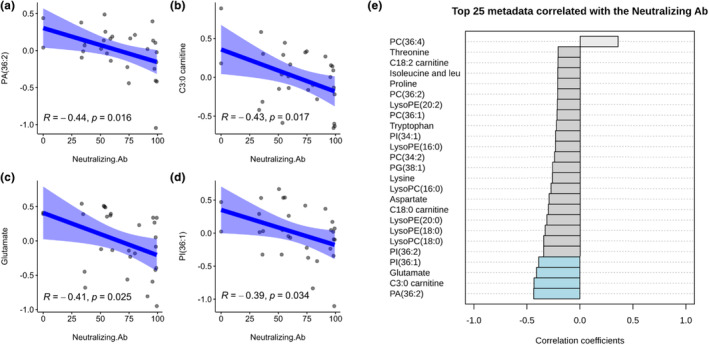
Correlation between plasma metabolomes and neutralizing antibody at 28‐day post vaccination. (a–d) Scatter plots of plasma metabolomes with statistically significant Pearson's correlation with neutralizing antibody (Log 10 transformation and Pareto scaling). (e) Top 25 plasma metabolomes correlated with neutralizing antibody.

When comparing the plasma metabolome at 28‐day after the complete course of each vaccine regimen to the baseline (Figure S4), only the AZ‐AZ regimen showed significant changes. Specifically, there was an upregulation of arginine (0.03), C10:1 carnitine (0.038). Conversely, there was a downregulation of lysoPE (18:0) (0.002), phenylalanine (0.003), lysoPC (18:0) (0.004), lysoPS (18:0) (0.010), aspartate (0.012), lysoPC (16:0) (0.014), lysoPE (20:0) (0.017), beta‐hydroxybutyrate (0.017), malate (0.026), histidine (0.042), isoleucine, and leucine (0.047).The correlations between postvaccination plasma metabolomes and the postvaccination nAb, for all regimens of vaccination, were analyzed (Figure 2). The results showed that postvaccination nAb was negatively correlated with PA (36:2) (Pearson's correlation coefficient = −0.44, *p*‐value = 0.016), C3:0 carnitine (−0.43, 0.017), glutamate (−0.41, 0.025), and PI (36:1) (−0.39, 0.034).

**FIGURE 3:**
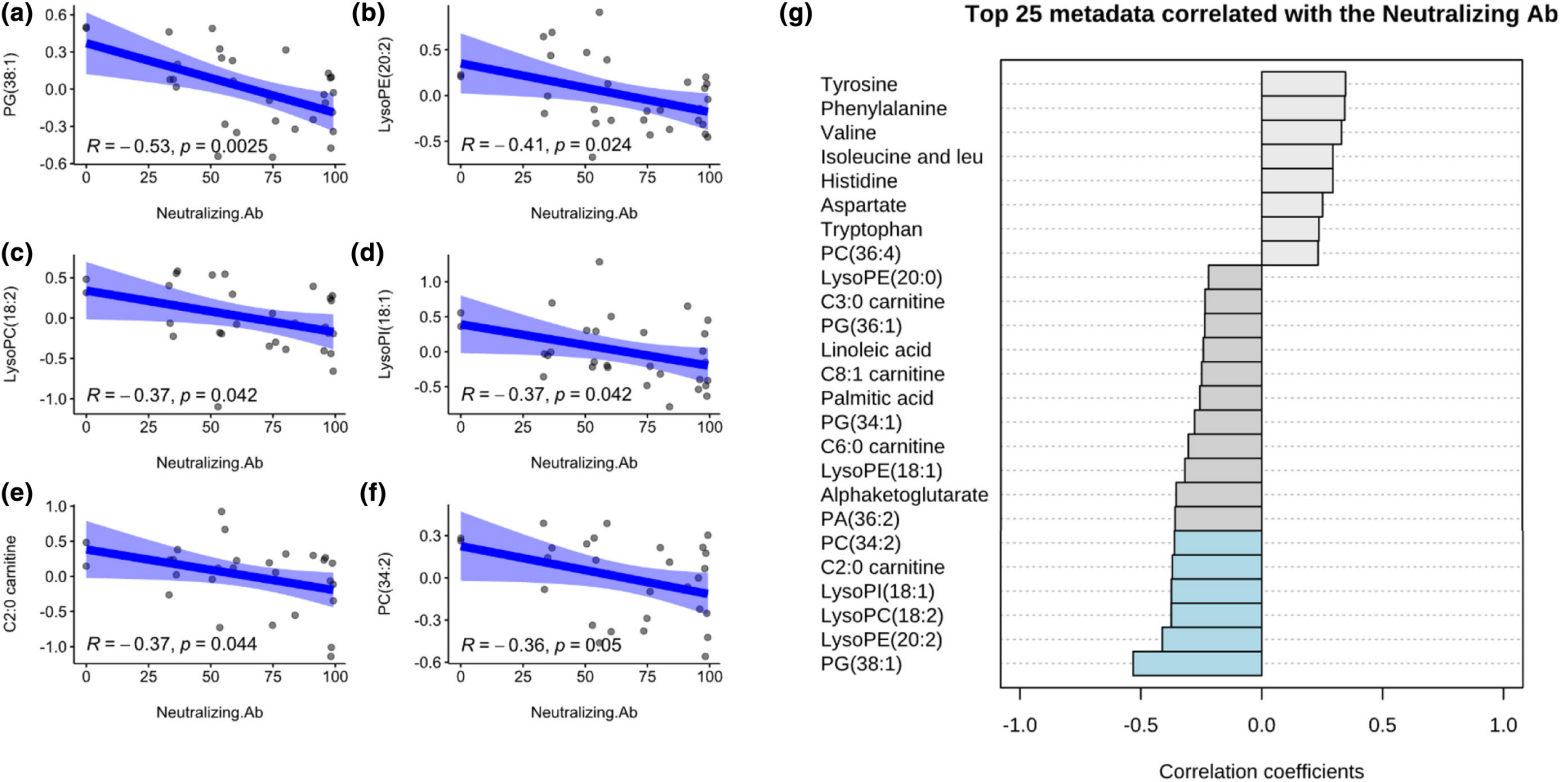
Correlation between plasma metabolomes at baseline and neutralizing antibody at 28‐day post vaccination (a–f) Scatter plots of plasma metabolomes with significant Pearson's correlation with neutralizing antibody (Log 10 transformation and Pareto scaling). (g) Top 25 plasma metabolomes correlated with neutralizing antibody.

In addition, the correlations of prevaccination plasma metabolomes and the postvaccination nAb were investigated in each regimen (Figure 3). The results also showed that down‐regulation of several plasma metabolomes were correlated with the higher level of nAb at the postvaccination phase, including PG (38:1) (Pearson's correlation coefficient = −0.53, *p*‐value = 0.002), lysoPE (20:2) (−0.41, 0.024), lysoPC (18:2) (−0.37, 0.042), lysoPI (18:1) (−0.37, 0.042), C2:0 carnitine (−0.37, 0.044), and PC (34:2) (−0.36, 0.05).

## 4 DISCUSSION

This study describes the initial in‐depth investigation into metabolomic shifts induced by diverse COVID‐19 vaccine regimens in patients receiving MHD. Our objectives were to unveil the potential underlying molecular basis of the immune response following COVID‐19 vaccination and to identify a metabolomics‐derived signature indication of the vaccination response. Notably, we observed distinct changes in plasma metabolome levels among various COVID‐19 vaccine regimens. Plasma metabolomic profiles in MHD patients from both pre‐ and post‐COVID‐19 vaccination revealed correlations with the vaccination response, as indicated by nAb levels. This investigation aimed to contribute valuable insights into the unique metabolic profiles associated with COVID‐19 vaccination in the specific context of MHD patients.

In the present study employing metabolomics to assess vaccine immunology, we identified distinct differences in metabolomic alterations after a complete course of vaccination among MHD patients. Significant alterations in several metabolites were noted. These findings imply that various COVID‐19 vaccination regimens elicit HMI through distinct pathways. Specifically, our results highlight the significance of amino acid and phospholipid metabolism, two critical metabolic pathways associated with vaccine‐induced immunogenicity (Perrin‐Cocon et al., 2006; Tomé, 2021). When comparing between each pair of vaccine regimens, AZ‐AZ and SV‐SV revealed the highest disparity in metabolomic changes, followed by SV‐AZ versus SV‐SV, and AZ‐AZ versus SV‐AZ, respectively. These variations were possibly attributed to several factors arising from differences in vaccine regimen and platform. AZD1222, a viral vector vaccine, employs a modified adenovirus to stimulate an immune response (Vanaparthy et al., 2021), while Sinovac, an inactivate virus vaccine, presents the immune system with viral proteins (Hu et al., 2023). The differences of these mechanisms may contribute to variations in metabolic responses. Humoral immune responses, particularly B cell activation and antibody production, are influenced by the specific antigens presented by the vaccines (Sadarangani et al., 2021). The observed downregulation and upregulation of metabolomes associated with humoral responses suggested a differential impact on B cell activation or antibody synthesis between the AZD1222 and Sinovac vaccines. This increased understanding of the metabolome changes sheds light on the distinct immunological responses elicited by these vaccine regimens in MHD patients.

The generation of effective antibodies postvaccination necessitates a complex interplay between humoral and cell‐mediated immunity (Chen et al., 2022). To ensure these optimal interactions, several essential substrates and an adequate energy supply are imperative. Upon scrutinizing each vaccine regimen, we observed the downregulation of some amino acids, and branched‐chain amino acid‐derived acylcarnitine. In contrast, there was an upregulation of glutamine. Aspartate and glutamate are crucial substrates for the synthesis of purine and pyrimidine nucleotides, essential for various immune cell proliferations, including lymphocytes (Tomé, 2021). Additionally, both these amino acids are linked to the citrate cycle, indicating their involvement in energy production (Tomé, 2021). The downregulation of these amino acids, in tandem with the upregulation of glutamine—a substrate of glutamic acid—suggests a heightened demand for these metabolomes in immune processes. Phenylalanine, which has been shown to be associated with humoral autoimmune diseases, implies an underlying role in the humoral response (Blackmore et al., 2020). Lysine plays a crucial role as an essential amino acid and a nutritional signal. There is substantial evidence to demonstrate that a dietary deficiency in lysine can hinder protein synthesis, including synthesis of cytokines, and the growth of lymphocytes, ultimately compromising the immune response (Kidd et al., 1997). Furthermore, research has indicated that insufficient dietary intake of lysine can diminish antibody responses and impair cell‐mediated immunity, as observed in studies involving chickens (Konashi et al., 2000). Phospholipid metabolism, including glycerophospholipids, and lysophosphatidylcholine, have been recognized as being involved in the modulation of dendritic cell generation. These are vital components and processes in innate and adaptive immune responses against viral infection and have a role in controlling the initiation of an adaptive immune response in vivo (Halder et al., 2019; Perrin‐Cocon et al., 2006). Collectively, these metabolomic alterations were likely to be associated with an increased demand for the activation and mounting of an immune response depending on individual vaccine regimens.

In comparing the metabolome alterations observed in MHD patients post‐COVID‐19 vaccination with those identified in the general population, we noted substantial similarities in most metabolomic changes. Specifically, both cohorts showed that alterations in certain amino acids, including glutamate and phenylalanine, play crucial roles in immune function (Dagla et al., 2022; He et al., 2022). Moreover, phospholipid changes were observed (Dagla et al., 2022), emphasizing a potential impact of COVID‐19 vaccination on lipid‐mediated immune processes. Nevertheless, distinctive metabolomic signatures in MHD patients, particularly unique alterations in bile acid metabolism, suggested that the interplay between vaccination and metabolic reprogramming may diverge in the context of renal impairment. These results implied that the primary alteration in the metabolomic profiles between MHD patients and the healthy population is comparable. However, these findings need further confirmation in a well‐designed study of a larger population utilizing the same vaccine regimen and platform, concurrently assessing vaccine immunogenicity over the same period.

In investigating the underlying molecular processes affecting the immune response following COVID‐19 vaccination, our study has uncovered intriguing associations between postvaccination plasma metabolomes and the subsequent nAb responses. Significantly, we have once again observed a negative association between glutamate and the immune response. Beyond its previously mentioned roles, glutamate plays an important role in the removal of oxidants and the regulation of the immune response through its intermediate precursor for glutathione synthesis (Self et al., 2004). Its negative association with nAb levels postvaccination implied that COVID‐19 vaccines might modulate the immune response through increased glutamate consumption to support higher energy demand and the sufficient activation and production of immune response. Additionally, the inverse correlation observed between PA (36:2), PI (36:1), and C3:0 carnitine levels and nAb highlighted the dynamic shifts in phospholipid and branched‐chain amino acid metabolism that occur following vaccination. PA (36:2), classified as a phosphatidic acid, and PI (36:1), a phosphatidylinositol, are glycerophospholipids that constitute the primary components of cell membranes across various cell types (Halder et al., 2019). Whereas, C3:0 carnitine is an intermediate metabolite of isoleucine, one of the amino acids integral to numerous immune processes (McCann et al., 2021). The negative association of these metabolomes suggests their essential role as precursors contributing to vaccine‐induced immune responses. These findings supported our earlier observations regarding the alterations in amino acid and phospholipid metabolisms following various COVID‐19 vaccine regimens.

The prediction of vaccine immunogenicity, particularly among individuals prone to a poor immune response, is crucial for identifying additional ways to enhance their immune function. Our study demonstrated that prevaccination plasma metabolomes, including PG (38:1), lysoPE (20:2), lysoPC (18:2), lysoPI (18:1), and PC (34:2), showed a negative correlation with the level of nAb at the postvaccination sampling time in MHD patients. These negative correlations suggested that alterations in phospholipid metabolism before vaccination may influence subsequent immune responses. These metabolomes could play a role in shaping the membrane environment of immune cells, influencing their functionality and responsiveness to vaccination. In addition, a previous study on an mRNA vaccine supported our findings revealing that lipoproteins and lipids, specifically polyunsaturated fatty acids, glycerophospholipids, and triacylglycerols, could discriminate between low and high responders even before the first vaccine dose (Dagla et al., 2022). Leverage of prevaccination metabolomic profiles for risk stratification and incorporating them into vaccination strategies represents a promising approach to enhancement of individualized care and will refine the design of future clinical trials. Continuous research and validation efforts will be essential to translate these insights into actionable clinical tools.

A limitation of our study was the relatively small size of the cohort and the absence of a healthy control group. Furthermore, this study did not include mRNA vaccines due to their unavailability in Thailand during the early stages of the COVID‐19 pandemic. At that time, the primary vaccines available were AZD1222 (AstraZeneca) and Sinovac. The observational nature of the cohort study also restricts the establishment of causality between identified metabolomes and nAb levels. Moreover, while variable matching could introduce selection bias, it helps harmonize factors that impact metabolomic profiles between groups. We acknowledge that our targeted metabolomic analysis focuses on a specific set of metabolites, which provides narrower coverage and limits our ability to explore potential novel compounds related to vaccine immunogenicity compared to an untargeted approach. However, our study only employed targeted metabolomics due to the well‐documented relevance of these metabolites to vaccine immunogenicity (Dagla et al., 2022; Diray‐Arce et al., 2020). Although untargeted metabolomics offers broader coverage, it often results in the identification of many unrelated or unknown metabolites. This complicates data interpretation and making it challenging to determine which pathways are directly associated with vaccine immunogenicity. The presence of numerous unidentified metabolites would require extensive validation and could divert focus from the key metabolic pathways of interest (Selamat et al., 2021). In light of these limitations, subsequent studies with larger sample sizes are required to overcome within‐group variability issues. These studies should also include other vaccine platforms, such as mRNA vaccines, and employ untargeted metabolomics approaches for broader metabolite coverage. The inclusion of a healthy control group and the incorporation of experimental models with in vitro and/or in vivo studies are also necessary steps for establishing a causal relationship between identified metabolomes and the response to COVID‐19 vaccination.

## 5 CONCLUSIONS

Distinct changes in plasma metabolomes were observed in MHD patients who received various vaccine regimens, indicating the unique interplay between individual vaccines and the immune system. Amino acid and phospholipid metabolisms played crucial roles in the molecular basis of nAb formation after COVID‐19 vaccination. Importantly, our analysis emphasized the significance of phospholipid metabolism as a predictive marker of the response to vaccination. Further investigations to validate these findings and their potential application in clinical practice are warranted.

## AUTHOR CONTRIBUTIONS

P.N. participated in participant enrollment, data analysis, data collection, data interpretation, and drafted the manuscript; S.C.C. designed the research, wrote the paper, gave critical comment and is the corresponding author. K.F. and N.W. participated in data analysis. C.T., S.S., W.N., and S.K. participated in all laboratory analysis. C.T., N.C. and S.K. participated in design, data interpretation, and revised manuscript critically for important intellectual content. All authors read and approved the final manuscript.

## FUNDING INFORMATION

This study was supported by the Research Grant for New Scholars from the Thailand Science Research and Innovation Program [grant number RGNS 64–059: C.T.]; the Research Grant from theNational Research Council of Thailand [grant number N42A650303: S.K.]; Chiang Mai University Fundamental Fund 2024 (S.K.); the NSTDA Research Chair Grant from the National Science and Technology Development Agency Thailand (N.C.); a Chiang Mai University Center of Excellence Award (N.C.); and the Distinguished Research Professor Grant from the National Research Council of Thailand [grant number N42A660301: S.C.C.].

## ETHICS STATEMENT

The study received approval from the local Institutional Review Board of the Faculty of Medicine, Chiang Mai University, Chiang Mai, Thailand (protocol code: MED‐2566‐0187, date of approval: September 29th 2023), in adherence with the Declaration of Helsinki.

## Supporting information


Figure S1.

## Data Availability

The metabolomics dataset generated and analyzed during the current study is available upon request of the corresponding author.
