# Supplementary material for: Alterations of plasma metabolomes and their correlations with immunogenicity in maintenance hemodialysis patients receiving different COVID‐19 vaccine regimens
Source: Physiol Rep. 2024 Aug 19;12(16):e70005. doi: 10.14814/phy2.70005 (PMC11333532; doi:10.14814/phy2.70005)
Supplement: Supplementary file 1 — Figure S1. [file PHY2-12-e70005-s001.docx]

**Figure legends of Supplementary figures**

**Supplementary Figure 1.** Study design and flow diagram of Immunogenicity and Safety of Homologous and Heterologous Prime-Boost of CoronaVac^®^ and ChAdOx1 nCoV-19 Among Hemodialysis Patients: An Observational Prospective Cohort Study.

**Supplementary Figure 2.** Principal component analysis of plasma metabolomes at baseline. AZ-AZ, homologous AZD1222 regimen; SV-AZ, heterologous Sinovac-AZD1222 regimen; SV-SV, homologous Sinovac regimen.

**Supplementary Figure 3.** Principal component analysis of plasma metabolomes at 28-day post vaccination. AZ-AZ, homologous AZD1222 regimen; SV-AZ, heterologous Sinovac-AZD1222 regimen; SV-SV, homologous Sinovac regimen.

**Supplementary Figure 4.** Comparison of plasma metabolomes at 28-day after the complete course of each vaccine regimen to baseline plasma metabolomes (A) AZ-AZ regimen, (B) SV-AZ regimen, and (C) SV-SV regimen.


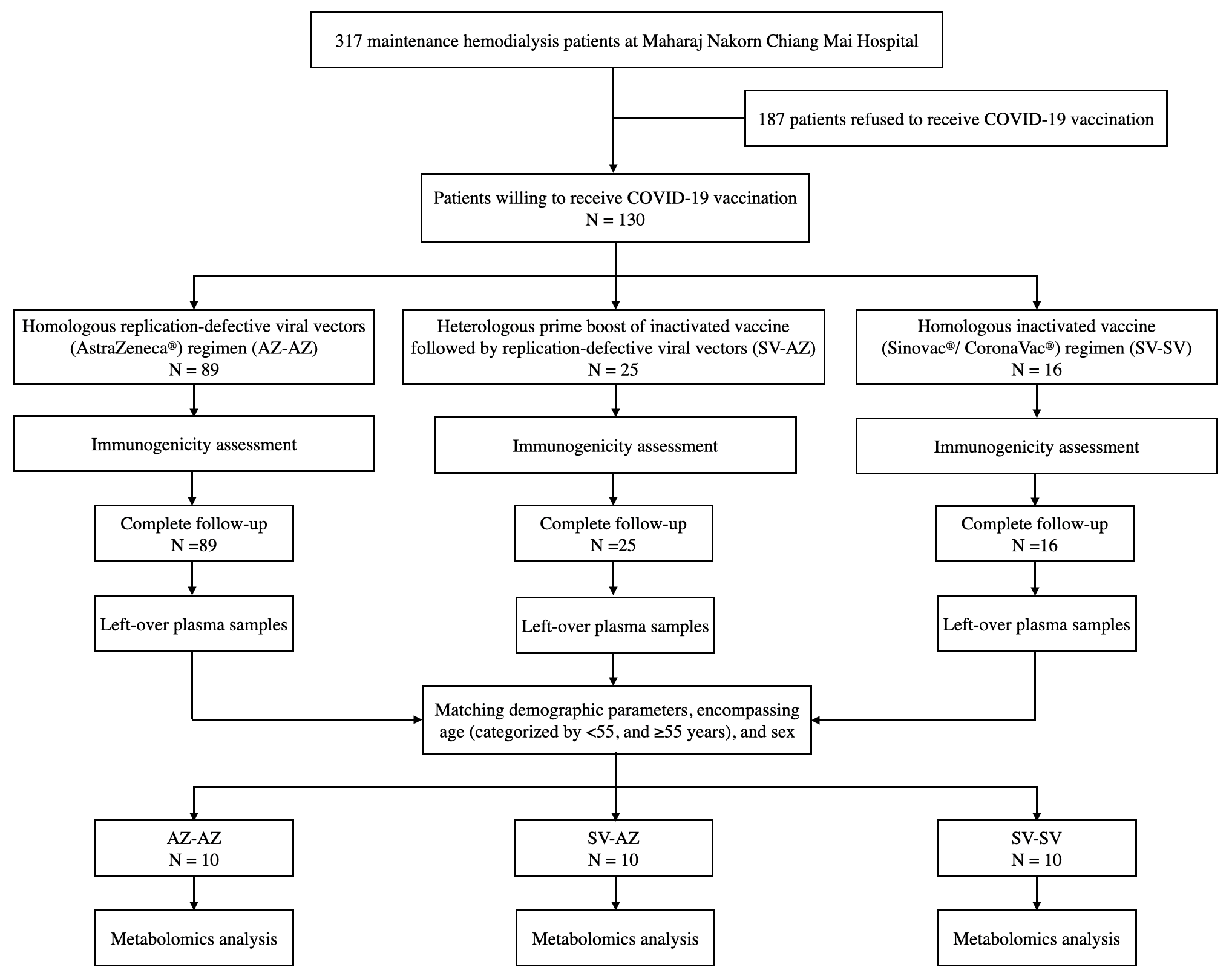


**Supplementary Figure 1.** Study design and flow diagram of Immunogenicity and Safety of Homologous and Heterologous Prime-Boost of CoronaVac^®^ and ChAdOx1 nCoV-19 Among Hemodialysis Patients: An Observational Prospective Cohort Study.


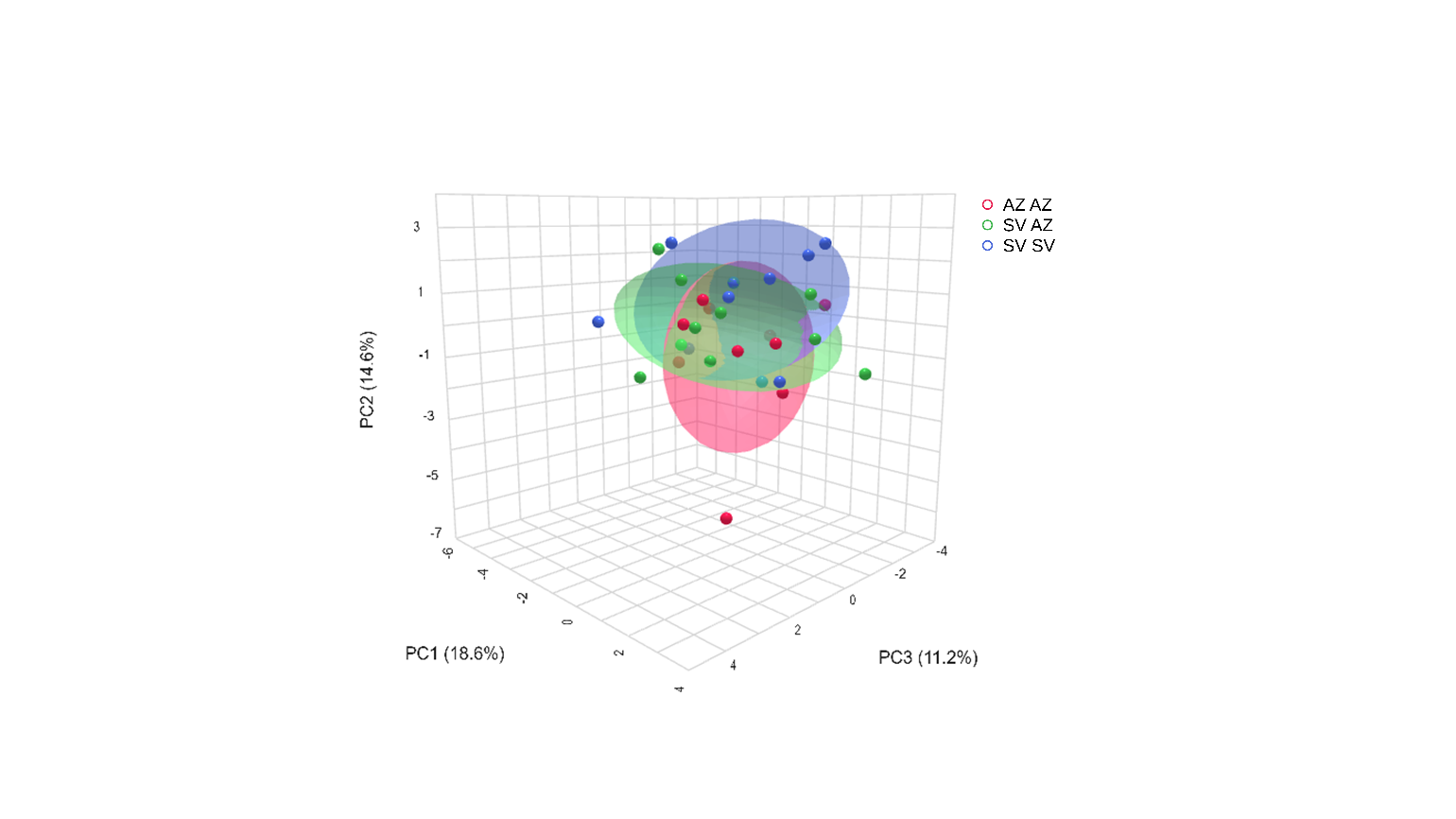


**Supplementary Figure 2.** Principal component analysis of plasma metabolomes at baseline. AZ-AZ, homologous AZD1222 regimen; SV-AZ, heterologous Sinovac-AZD1222 regimen; SV-SV, homologous Sinovac regimen.


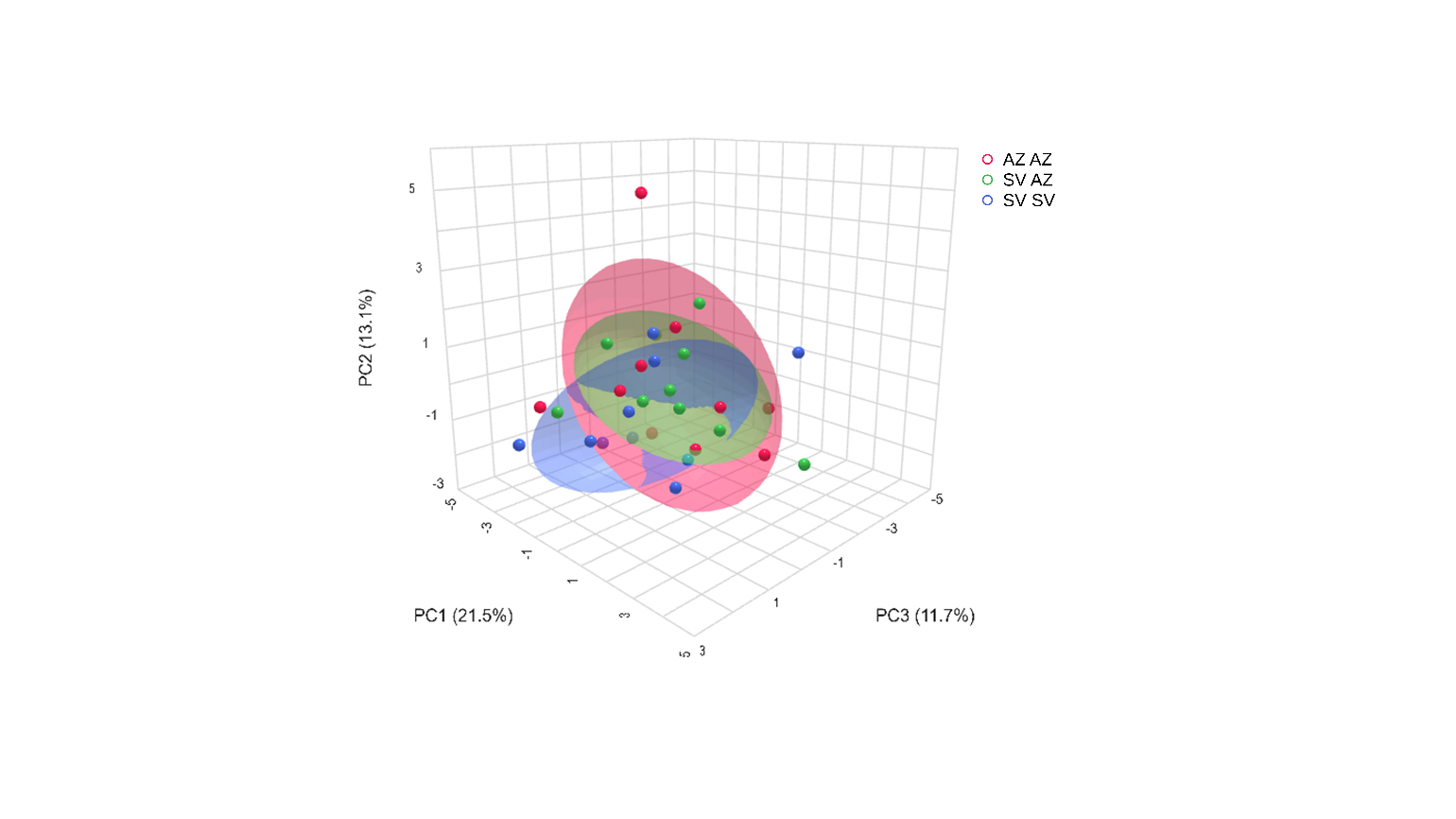


**Supplementary Figure 3.** Principal component analysis of plasma metabolomes at 28-day post vaccination. AZ-AZ, homologous AZD1222 regimen; SV-AZ, heterologous Sinovac-AZD1222 regimen; SV-SV, homologous Sinovac regimen.


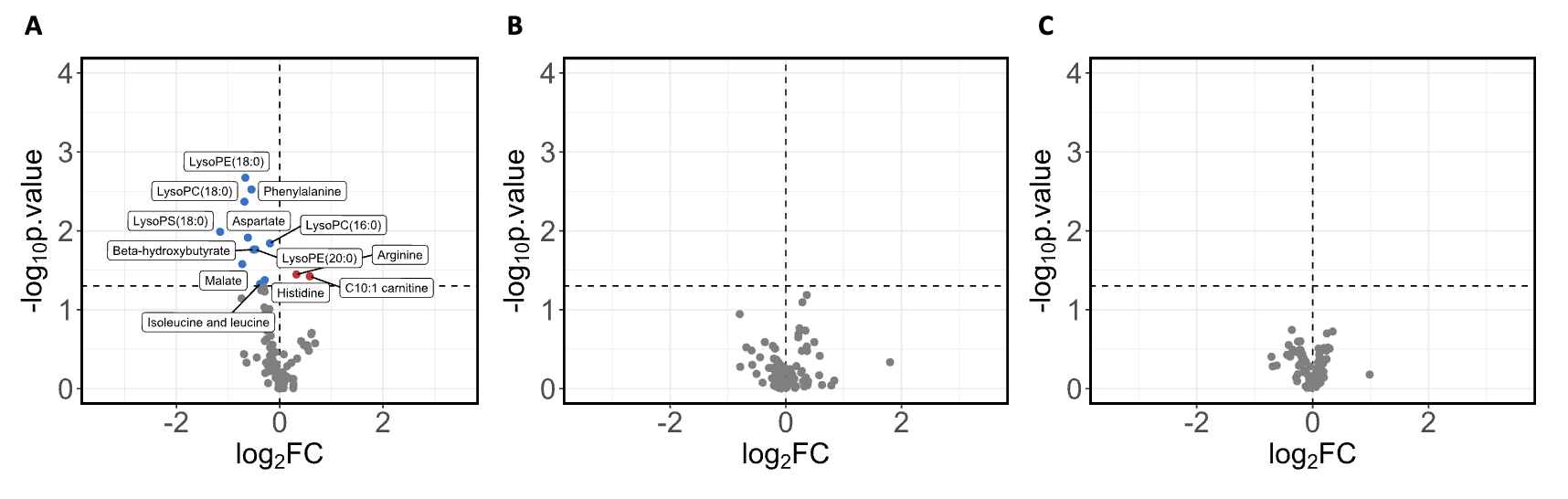


**Supplementary Figure 4.** Comparison of plasma metabolomes at 28-day after the complete course of each vaccine regimen to baseline plasma metabolomes (A) AZ-AZ regimen, (B) SV-AZ regimen, and (C) SV-SV regimen.
